# Incidence of Pyogenic Arthritis in Aragón, Spain, From 2015 to 2023

**DOI:** 10.7759/cureus.99385

**Published:** 2025-12-16

**Authors:** Alberto San Pedro, Elena García-Cristobal, Felicito Garcia-Alvarez

**Affiliations:** 1 Orthopaedic Surgery and Traumatology, Hospital Clinico Universitario Lozano Blesa, Zaragoza, ESP; 2 Orthopaedics and Traumatology, Universidad de Zaragoza, Zaragoza, ESP; 3 Orthopaedics and Traumatology, Hospital Clinico Universitario Lozano Blesa, Zaragoza, ESP; 4 Orthopaedics and Traumatology, Instituto de Investigaciones Sanitarias de Aragón, Zaragoza, ESP

**Keywords:** age distribution, comorbidities, epidemiology, musculoskeletal infection, pyogenic arthritis

## Abstract

Aim

The main objective of our study is to analyze the evolution of hospitalizations coded M00.9 (pyogenic arthritis) in Aragón (Spain) since 2015.

Methods

This is an observational, descriptive, and analytical study analyzing cases coded as pyogenic arthritis (M00.9), according to the International Classification of Diseases, version 10 (ICD-10), in both public and private hospitals in the Autonomous Community of Aragón between 2015 and 2023. Primary data were obtained from Aragón’s Health Big Data (BIGAN), linked to the Aragón Health Sciences Institute, and the data were analyzed by us, considering a p-value <0.05 as statistically significant.

Results

A total of 261 patients with pyogenic arthritis were identified. The incidence of pyogenic arthritis showed an increasing trend, with an incidence of 1.76/100,000 inhabitants in 2016 and 1.36/100,000 inhabitants in 2021, which rose to 5.65/100,000 inhabitants in 2022, followed by a decrease in 2023 without reaching the previous levels of 2021. Men accounted for 160 (61.3%) of the 261 patients with pyogenic arthritis, while 101 were women (38.7%). The mean age was 54.96±29.21 years, with no significant age differences between men and women (men 54.8±27.3; women 55.22±32.14). Among zero- to four-year-old children, there were 35 cases (13.41%) (18 boys and 17 girls). There were 119 cases (45.59%) in patients aged >65 years (66 men, 55.46%). In 2016, the incidence was 3.41 cases/100,000 persons aged zero to four years, and 2.87 cases/100,000 persons aged >65 years; in 2022, it was 8.04 cases/100,000 persons aged zero to four years and 12.97 cases/100,000 persons aged >65 years. The monthly distribution did not show significant differences; the highest incidence was recorded in August (28 patients) and September (31 patients). A total of 90.2% of pyogenic arthritis cases were treated in the public health system. The mean length of hospital stay was 13.03±14.53 days. Readmission occurred in 23.37% of patients with pyogenic arthritis. The most frequent comorbidities were: chronic hematologic disease (37.4%), hypertension (35.11%), dyslipidemia (29.01%), rheumatologic disorder (24.05%), mental disorder (20.23%), diabetes (19.85%), and vascular disease (19.47%).

Conclusions

In 2022, there was a significant increase in pyogenic arthritis compared to previous years, with a decrease in 2023 that did not return to the levels prior to 2021. There was a predominance of pyogenic arthritis in men.

## Introduction

Septic arthritis is defined as an infection of the joint space and the surrounding synovial membrane, which may result in cartilage destruction, impaired joint function, and accelerated osteoarthritis [1,2]. Musculoskeletal infections represent a significant cause of disability worldwide. Within 15 years after septic arthritis, 8.7% patients had received arthroplasty, corresponding to an annual risk of arthroplasty that was about six times that of the general population [3]. Several risk factors have been identified, including advanced age, diabetes mellitus, rheumatoid arthritis, and the increasing use of immunosuppressive therapies; in addition, local factors such as skin infections, cutaneous ulcers, and the growing number of invasive procedures - particularly prior intra-articular injections - are also associated with its development [4,5].

Some studies included periprosthetic infections under septic arthritis, which presented a variable incidence of 10.2/100,000 in the year 1995 to 3.1/100,000 in the year 2000 [6]. A study published in 2020 reported an incidence rate of 13 cases per 100,000 person-years in large joints [7]. In patients with concomitant *Staphylococcus aureus* bacteremia, a 2022 review highlighted a poorer prognosis, with reported mortality rates exceeding 50% [8]. Differences about incidence have been related with ethnic variation [7]. Incidence rises with age and socioeconomic deprivation [7]. Large joints are more frequently affected than small joints, with the knee being the most commonly involved large joint, whereas in small joints, the interphalangeal joints of the hand predominate [5-7,9,10]. The most common etiology is hematogenous seeding of the synovial tissue, although it may also result from the extension of metaphyseal osteomyelitis, surgical procedures, or direct traumatic inoculation [2,4,5,11]. Septic arthritis is generally due to monomicrobial infections, with polymicrobial cases accounting for fewer than 10% [4]. Most cases are caused by Gram-positive bacteria, with *S. aureus* being the leading pathogen, responsible for more than half of the cases [4,6,7], followed by various types of *Streptococcus *species in more than 10% of cases [4,7]. Gram-negative bacteria account for approximately 10%-19% of infections [4,7]. The type of causative organism may vary depending on patient characteristics and type of articulation [4,12].

Several factors contribute to the epidemiological changes of infectious diseases, migration, increased travel, and global trade allow outbreaks to spread from remote areas to the rest of the world within days; furthermore, high-density living environments facilitate the transmission of infectious diseases in urban settings, and climate change has also been identified as a contributor to shifts in transmission patterns of infectious diseases [13-16]. In the last decade, the COVID-19 pandemic significantly altered travel patterns and social behavior, and it is therefore plausible that it also influenced the epidemiology of septic arthritis. We have not found any study about post-COVID-19 epidemiology of septic arthritis.

The primary objective of our study is to analyze the epidemiological evolution and incidence of hospitalizations coded M00.9 (pyogenic arthritis) in Aragón (Spain) from 2015 to 2023, specifically detailing its distribution by sex and age group and the prevalence of associated comorbidities.

## Materials and methods

Ethical considerations

The project was approved by the Research Ethics Committee of the Autonomous Community of Aragón (CEICA; reference 8PI24-170). The study was conducted using anonymized data from a database devoid of personal identifiers; patients included in the study were identified solely by a case number (without medical record number or initials), ensuring full confidentiality. Patient consent was waived due to anonymization.

Study design and population

An observational, descriptive, analytical, and cross-sectional study was conducted, analyzing cases coded as septic arthritis according to International Classification of Diseases (ICD) 10 codes in both public and private hospitals of the Autonomous Community of Aragón (Spain). The population of Aragón on January 1, 2022 was 1,326,315 inhabitants (49,782 aged zero to four years; 292,934 aged ≥65 years). Previous census figures were: 1,326,261 in 2021, 1,329,391 in 2020, 1,319,290 in 2019, 1,308,728 in 2018, 1,308,750 in 2017, 1,308,563 in 2016 (58,599 aged zero to four years; 278,361 aged ≥65 years), and 1,317,847 in 2015 [17].

For sample size, data were obtained from the Aragón Health Big Data (BIGAN) system, managed by the Aragón Institute of Health Sciences. All cases coded as unspecified pyogenic arthritis (M00.9) according to ICD-10, from 2015 to 2023, were requested from BIGAN. The system provided the raw data of all cases meeting the inclusion criteria. The inclusion criteria were having been coded as M00.9 according to ICD-10 from 2015 to 2023 in Aragon. The exclusion criteria were not to having been coded as M00.9 according to ICD-10 from 2015 to 2023 in Aragon. A total of 261 patients diagnosed with pyogenic arthritis were identified. The study included both residents and non-residents of Aragón, holding a health card from Aragón or other autonomous communities, who were treated in any hospital in Aragón, regardless of whether the institution was public or private. Patients from healthcare insurances General Mutual Fund of Civil Servants of the State (MUFACE), General Legal Mutual Fund (MUGEJU), and the Social Institute of the Armed Forces (ISFAS) were considered as privately funded.

All data were obtained in a coded format using numeric values; at no time were personal identifiers such as names, surnames, or medical record numbers available. This ensured full protection of patient anonymity throughout the study.

Selection and definition of variables

Several variables were analyzed, including: year of diagnosis (2015-2023), month of diagnosis, patient sex (male/female), patient age, type of healthcare facility attended (public/private), and need for hospital readmission. Readmission was considered any admission during the 30 days following the initial admission and that is motivated by the same cause or a complication of the same. The presence of comorbidities was also recorded (Table 1).

**Table 1:** Comorbidities International Classification of Diseases, version 10 (ICD-10) codes.

Condition	ICD-10 Codes
Asthma	J45
Autoimmune disease	E06.9, M35.3, L40.9, M32.9
Bronchitis	J40
Cancer	C00-D49
Cerebrovascular disease	I60-I69
Chronic heart disease	I25.9, I48.9
Chronic hematologic disease	D50-D77
Chronic kidney disease (CKD)	N18.9
Chronic obstructive pulmonary disease (COPD)	J44.9
Dementia	F03.9
Diabetes	E08-E13
Dyslipidemia	E78.9
Embolism	I63.9
Heart failure	I50.9
Patients infected with the human immunodeficiency virus (HIV)	B20
Hypertension (HTN)	I10
Immunodeficiency	D89.9
Leukemia	C91-C95
Liver disease	K76.9
Malignant lymphoma	C81-C90
Mental disorder	F01-F99
Myocardial infarction (MI)	I21.9
Neurological disorder	G00-G99
Obesity	E66.9
Rheumatologic disorder	M15-M19
Smoking	F17.21
Ulcer disease	L89, L97
Vascular disease	I70-I79

Statistical analysis

Statistical analysis was performed using Statview-Statgraphics software version 5.0.1 (SAS Institute Inc., Cary, NC, USA). The chi-square test and Fisher’s exact test were applied for the comparison of qualitative variables. To compare the variation in incidence between different years, a linear regression with Snedecor's F was applied. All data are presented as means, with standard deviations reported as measures of variability. A p-value <0.05 was considered statistically significant.

## Results

**Figure 1:**
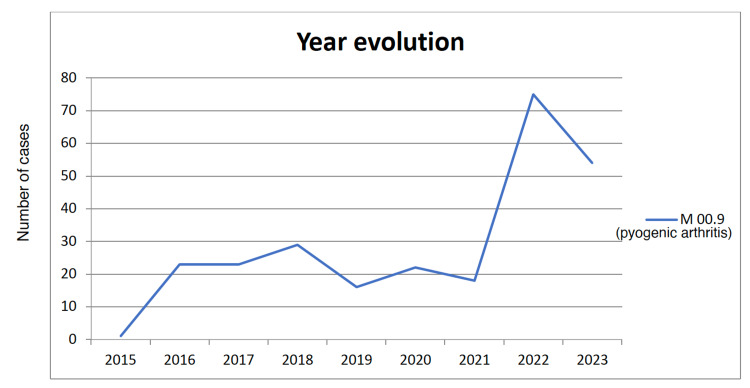
Number of cases of pyogenic arthritis (M00.9) in Aragon by year between 2015 and 2023

We identified 261 cases coded as M00.9 between 2015 and 2023. A rising trend was observed (Figure 1), reaching a peak in 2022 with 75 patients (28.7% of the total). Regression analysis showed significant variation (R²=0.494, F=6.84, p=0.0346 ). The year 2022 showed an increase compared with 2021 (18 patients). In 2023, the number of cases decreased to 54 patients (20.7% of the total), and case numbers did not return to 2021 levels or those of previous years.

The incidence was 1.76 per 100,000 inhabitants in 2016, 1.36 per 100,000 in 2021, and increased to 5.65 per 100,000 in 2022 (Table 2). Of the 261 patients with pyogenic arthritis, 160 were men (61.3%) and 101 were women (38.7%).

**Table 2:** Incidence by year and age group.

Year	Population	Incidence case/100,000 inhabitants
2016	General	1.76
2016	0-4 years old	3.41
2016	>65 years old	2.87
2021	General	1.36
2022	General	5.65
2022	0-4 years old	8.04
2022	>65 years old	12.97

**Figure 2:**
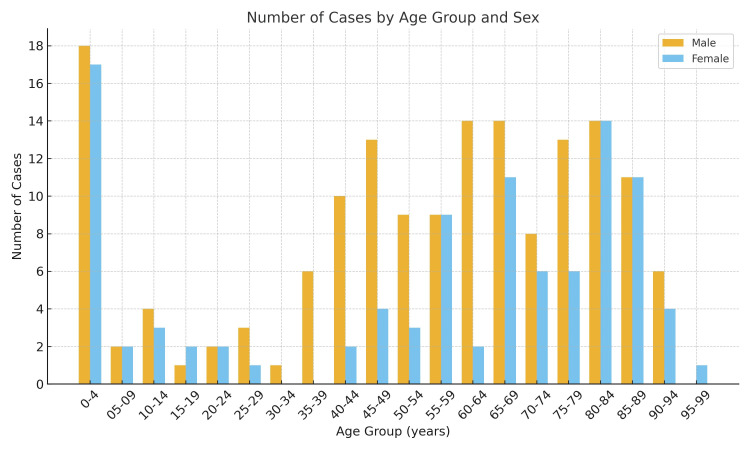
Cases by age and sex.

The mean age of patients was 54.96±29.21 years, with no significant differences between men (54.8±27.3 years) and women (55.22±32.14 years). The chi-square test showed significant differences in the distribution of cases by age group (p=6.42×10⁻²³), with a peak of 35 cases (13.41%) (18 men and 17 women) in early childhood, between zero and four years of age (Figure 2).

Across all study years, 119 cases (45.59%) occurred in patients aged ≥65 years (66 men, 55.46%; 53 women, 44.54%). In 2016, there were two cases among children aged zero to four years (3.41 per 100,000 population aged zero to four years) and eight cases among individuals ≥65 years (2.87 per 100,000 population aged ≥65 years) (Table 2). In 2022, there were four cases in the zero- to four-year age group (8.04 per 100,000 population aged zero to four years) and 38 cases in patients ≥65 years (12.97 per 100,000 population aged ≥65 years) (Table 2).

**Figure 3:**
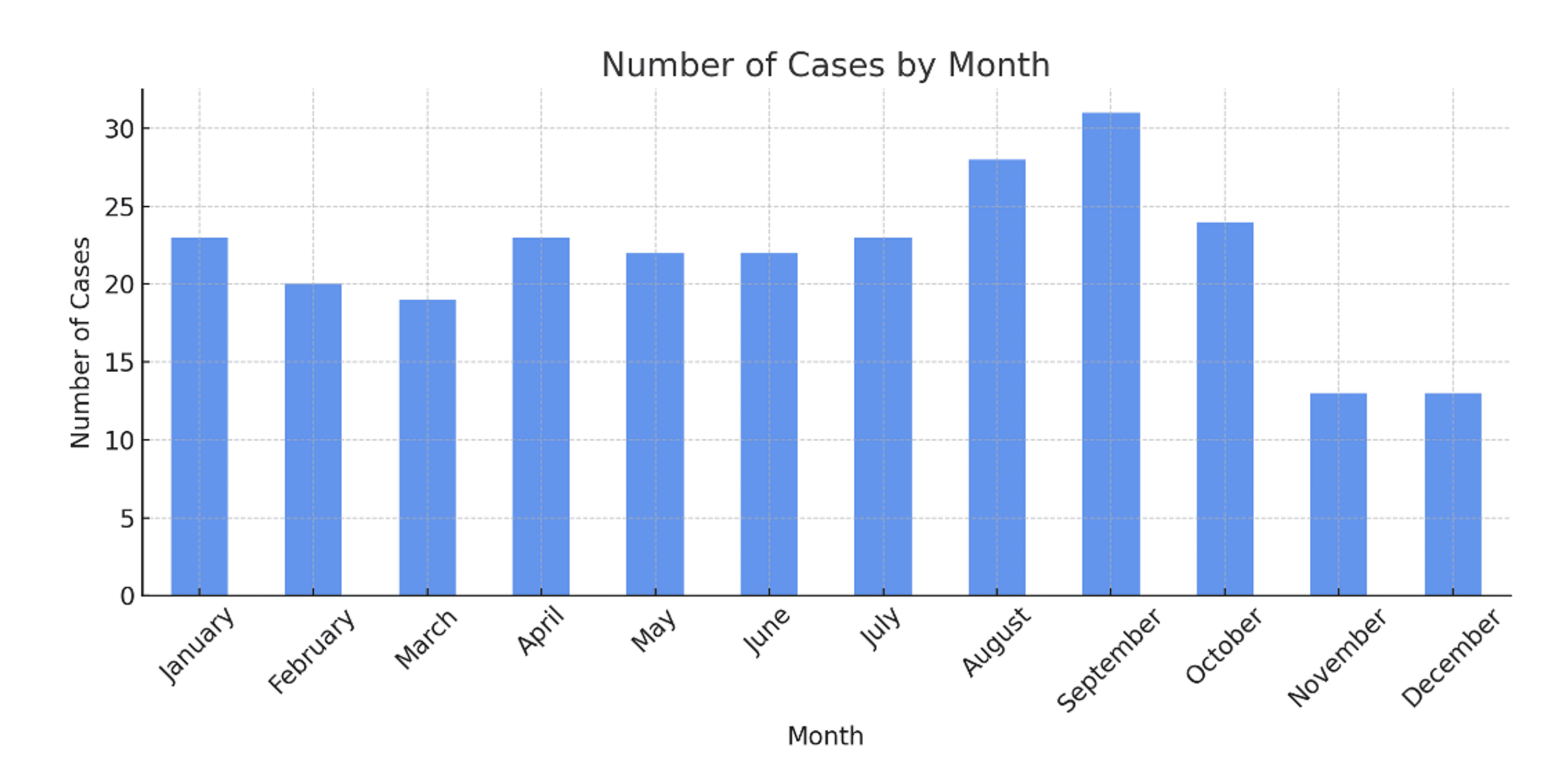
Number of cases by month.

When analyzing the monthly distribution of cases (Figure 3), a progressive increase was observed from spring through summer, reaching a peak of incidence in August (28 patients) and September (31 patients, 11.88% of the total), followed by a gradual decline during the winter months. However, no statistically significant differences were found between months.

Regarding type of healthcare, 90.2% of pyogenic arthritis cases were managed within the public healthcare system, while only 9.8% were treated in private facilities. The mean length of hospital stay was 13.03±14.53 days; by sex, it was 13.25±15.56 days for men and 12.8±12.54 days for women, with no significant differences. A total of 23.37% of patients were readmitted, including 39 men and 22 women, although this difference was not statistically significant.

The most frequent comorbidities associated with pyogenic arthritis (Table 3) were: chronic hematological disease (37.4% of patients), hypertension (35.11%), dyslipidemia (29.01%), rheumatologic disorders (24.05%), and mental health conditions (20.23%).

**Table 3:** Comorbidities in patients with pyogenic arthritis (M00.9).

Comorbidity	Percentage (%)	Number of Cases
Chronic hematologic disease	37.40	98
Hypertension	35.11	92
Dyslipidemia	29.01	76
Rheumatologic disorder	24.05	63
Mental disorder	20.23	53
Diabetes	19.85	52
Vascular disease	19.47	51
Chronic heart disease	17.56	46
Cancer	12.98	34
Neurological disorder	12.21	32
Smoking	11.83	31
Embolism	10.31	27
Obesity	9.54	25
Autoimmune disease	9.16	24
Chronic kidney disease	8.78	23
Chronic obstructive pulmonary disease (COPD)	5.73	15
Heart failure	5.34	14
Cerebrovascular disease	4.58	12
Dementia	3.05	8
Liver disease	2.29	6
Leukemia	1.91	5
Human immunodeficiency virus (HIV) infection	1.15	3
Myocardial infarction	0.76	2
Ulcer disease	0.76	2
Immunodeficiency	0.38	1
Malignant lymphoma	0.38	1

## Discussion

In this study, we reviewed data from patients diagnosed with pyogenic arthritis between 2015 and 2023 in Aragón, using raw data obtained from the Aragón Health Big Data system (BIGAN). We observed a significant increase in diagnoses in 2022 compared with 2021, followed by a decrease in 2023, although incidence did not return to pre-2021 levels. Studies from other countries have similarly reported an increase in the incidence of septic arthritis in recent years, although incidence figures may vary widely across regions and time periods within the same country. For example, McBride et al., in a study based on patients admitted with septic arthritis between 2009 and 2014 at a hospital in Auckland, New Zealand, reported an incidence of 21 per 100,000 inhabitants, higher than the 12 per 100,000 reported by Kennedy et al. between 2009 and 2013 in a hospital in Christchurch, New Zealand [4-7]. McBride et al. included cases with codes M00.0 (staphylococcal arthritis), M00.1 (pneumococcal arthritis), M00.2 (streptococcal arthritis), M00.8 (unspecified bacterial arthritis), and M00.9 (unspecified pyogenic arthritis). We only included the generic code M00.9. Geirsson et al. documented 253 cases of septic arthritis M00.9 in Iceland between 1990 and 2002, with the annual incidence rising from 4.2 per 100,000 in 1990 to 11 per 100,000 in 2002. In comparison, the incidence in our study was lower than in these reports (1.76 per 100,000 in 2016 and 5.65 per 100,000 in 2022) [18].

The COVID-19 pandemic drastically altered the landscape of infectious diseases worldwide, posing a major challenge for healthcare systems and professionals [19]. Several European countries (including Ireland, France, the Netherlands, Sweden, and the United Kingdom) reported an increase during 2022 - particularly from September onwards - in cases of invasive group A *Streptococcus* (iGAS) among children under 10 years of age [20]. In a 2023 study conducted in a Spanish hospital, Maldonado-Barrueco et al. [21] documented an increase in the incidence of invasive *Streptococcus pyogenes* infections between January and May 2023 compared with previous years. The World Health Organization [22] also reported a significant rise in invasive *S. pyogenes* infections in Sweden, with 220 cases notified between June 2021 and June 2022 compared to 173 cases in the same period of 2020-2021; and in Ireland, 23 cases were reported between October and December 2022 compared with 11 cases in the same period of 2019, before the pandemic.

Not only did septic arthritis increase in 2022, but other types of infections also showed an increase that year. In fact, in October 2022, several European countries also reported a rise in pediatric hospitalizations due to RSV, along with other respiratory viruses such as influenza and SARS-CoV-2 [20]. Since early 2022, many countries have experienced a tenfold increase in infectious disease incidence compared with pre-pandemic levels [19]. This surge has been partly attributed to decreased vaccination coverage during the pandemic, particularly against diphtheria, tetanus, and pertussis in 2021. Moreover, restrictions on population movement may have altered collective immunity. In Spain, a state of alarm was declared on March 14, 2020, restricting free movement, with strict confinement measures gradually eased from April 28, 2020. Social distancing measures and widespread mask use reduced the circulation of common pathogens, the subsequent resurgence of infections may be explained by decreased herd immunity during confinement and, consequently, increased population vulnerability once normal social interactions resumed. Indeed, influenza cases increased after the pandemic, compared with 2019, by 75% in Europe and 28% in the United States [19].

In our study, men accounted for 61% of pyogenic arthritis cases, although no sex-related differences were observed among patients aged zero to four years. A Spanish study covering the 2010-2019 period reported that men represented 62.8% of cases [10]. Similarly, Kennedy et al. found that 66.9% of patients were men (166/248) versus 33.1% women (82/248) [4]. Geirsson et al. included 253 cases, of which 62.85% were men.

In our series, the mean age of patients with pyogenic arthritis was 54.96 years, with no differences between men and women [18]. We found differences in case distribution by age group, with a peak of 13.41% of cases in children aged zero to four years and another peak of 45.59% in patients aged ≥65 years. The incidence in these age groups was higher than the general age-specific incidence, rising from 3.41 cases per 100,000 in children aged zero to four years in 2016 to 8.04 per 100,000 in 2022; and from 2.87 per 100,000 in older adults (≥65 years) in 2016 to 12.97 per 100,000 in 2022. Ryan et al. [23] also reported that septic arthritis cases were more frequent in children under 10 years (12.7% of cases) and in adults over 60 years (54.7% of cases).

We found no evidence in the literature demonstrating a significant seasonal variation in the distribution of infections, nor did our results reveal such differences.

With regard to healthcare setting, 90% of septic arthritis cases in our study were managed in the public system, compared with 10% in private institutions. According to data from the Spanish National Institute of Statistics (INE) for 2017 [24], 16.3% of the national population had private healthcare coverage, compared with 16.07% in Aragón. One might assume that diagnostic coding was not systematically performed in private hospitals. However, in a recent study by our group on shoulder arthroscopy - using the same BIGAN database covering 2015-2023 - we found that, in some years, case registration was actually higher in the private sector than in the public sector.

Among the comorbidities analyzed in our study, chronic hematological diseases predominated, including anemias, thrombocytopenias, neutropenias, pancytopenias, and coagulation disorders such as antiphospholipid syndrome. The cumulative incidence of septic arthritis was higher in the iron-deficiency anemia cohort than in the control cohort in the Taiwanese longitudinal health insurance database, iron-deficiency anemia patients had a 2.53-fold risk of septic arthritis compared to control subjects [25]. Also, some independent risk factors for periprosthetic joint infection have been detected, in decreasing order of significance: congestive heart failure, chronic pulmonary disease, preoperative anemia, diabetes, depression, renal disease, pulmonary circulation disorders, obesity, rheumatologic disease, psychoses, metastatic tumor, peripheral vascular disease, and valvular disease [26]. In the study by McBride et al. on septic arthritis, the most common comorbidities were current smoking (35%), osteoarthritis (29%), diabetes mellitus (24%), gout (15%), and rheumatoid arthritis (2%) [7].

A clinical study would be necessary to analyze the causative microbiological agents, as well as their possible relationship with different comorbidities and age groups.

Limitations

The study has several limitations. The methodology did not involve a detailed review of individual clinical records, which could have provided additional information such as culture results, antibiotic sensitivities, or the presence of fever. The coders assigned code M00.9 to the included cases based on hospital discharge reports. While we were unable to access the microbiological culture results, the clinical staff who treated these patients and established the diagnosis in the discharge report did have access to those microbiological results. Furthermore, the study depends on accurate coding within participating hospitals; however, coding is typically performed by trained professionals specialized in this task. The use of data from 2015 onward is explained by the transition within BIGAN from ICD-9 to ICD-10 coding systems implemented that year.

## Conclusions

In 2022, there was a significant increase in cases of pyogenic arthritis (encoded M00.9) compared with previous years, followed by a decrease in 2023, although incidence did not return to pre-2021 levels. In our study, men were more frequently affected than women. Patients with pyogenic arthritis also presented with a high prevalence of comorbidities.
